# Prevalence and Risk Factors of Dance Injury During COVID-19: A Cross-Sectional Study From University Students in China

**DOI:** 10.3389/fpsyg.2021.759413

**Published:** 2021-10-27

**Authors:** Yanan Dang, Yiannis Koutedakis, Rouling Chen, Matthew A. Wyon

**Affiliations:** ^1^School of Sport, University of Wolverhampton, Wolverhampton, United Kingdom; ^2^Functional Architecture of Mammals in Their Environment (FAME) Laboratory, University of Thessaly, Trikala, Greece; ^3^National Institute of Dance Medicine and Science, Birmingham, United Kingdom

**Keywords:** injury prevalence, risk factors, ballet, Chinese dance, pre-professional training

## Abstract

**Objectives:** Although coronavirus disease 2019 (COVID-19) has transformed the training environment of dancers worldwide, little is known on how this has affected injury prevalence, causes, and risk factors.

**Methods:** An online investigation involving Chinese full-time dance students was conducted (September–November 2020), which covered two 6-month periods just before and during the first COVID-19 lockdown.

**Results:** 2086 students (19 ± 2.4 years) responded to the investigation. Injury prevalence dropped from 39.6% (before the lockdown) to 16.5% (during the lockdown) (*p* < 0.01). It was noted that a significant increase in injury severity during the lockdown was caused due to a 4.1% increase in moderate-to-severe injuries (*p* < 0.05). During the lockdown, the injuries on the lower back, feet, and shoulders decreased significantly (*p* < 0.01), but the knee, ankle, and groin/hip joint injuries remained the same. Fatigue and the recurrence of an old injury remained as the top two perceived causes of an injury between the two periods with the increase in an unsuitable floor (*p* < 0.01), a cold environment (*p* < 0.05), and set/props (*p* < 0.05). The fatigue degree of students decreased (*p* < 0.01) and their hours of sleep increased (*p* < 0.01) during the lockdown. Binary Logistic Regression analysis indicated that dance injury is associated with fatigue, the hours of sleep, and the actions taken if they suspect an injury during the lockdown (*p* < 0.05), but is only related to the time set aside for a cooldown and age before the lockdown period (*p* < 0.05).

**Conclusion:** Although injury prevalence dropped significantly during the first COVID-19 lockdown in Chinese dance students, the main dance injury characteristics remained the same. Decreased fatigue and longer hours of sleep could explain the aforementioned drop in injury prevalence during the lockdown.

## Introduction

The arrival of the coronavirus disease 2019 (COVID-19) forced nearly all pre-professional dancers to train and study from home due to the lockdown. Prior to this, the reported injury prevalence for dancers was between 3 and 95% (Brinson and Dick, 1996; Laws and Apps, 2005; Hincapié et al., 2008; Riding McCabe et al., 2014; Dang et al., 2020; Henn et al., 2020; Uršej and Zaletel, 2020), with an injury incidence between 0.1 and 4.9 per 1,000 h (Hopper et al., 2014a; Bronner and Bauer, 2018; Bronner et al., 2018; Kenny et al., 2018; Brooker, 2020; Stephens et al., 2021). Injuries were mainly in the muscles of the lower limb and chronic in nature (Allen et al., 2013; Dang et al., 2020). Perceived causes of these injuries were overwork, fatigue, and the recurrence of an old injury (Dang et al., 2020).

In China, dance students in full-time training are either university students (4-year course) (Beijing Dance Academy, 2021; Beijing Normal University, 2021) or pre-professional secondary school students (6-/7-year course) (Academy TASSoBD, 2021). Universities recruit dance students from pre-professional secondary schools with full-time training or high-school students with part-time training. Pre-professional secondary schools recruit students (11–12 years old) from primary schools or junior high schools (Academy TASSoBD, 2021). Pre-professional training usually starts around the age of 11–12 years with students training 11–17 h a week until they are 18 years old, and the same training at a university (18–22 years old), the hours increased to between 17 and 20 h a week.

Chinese students can major in Dancology (this is a general full-time course that includes training in Basic Ballet Training, Chinese Classic Dance, Chinese Folk Dance, Contemporary Dance, Dance Choreography, and Dance Theory), Chinese Classical Dance (sole specialization at a conservatoire), Chinese Folk Dance (sole specialization at a conservatoire), or Chinese Dance (a combination of classical and folk dance usually at a regular university).

Recent studies that have incorporated the lockdown period have focused on how dance can help the general population stay fit and healthy (Finahari and Rubiono, 2020; Zhang, 2020; Lotan Mesika et al., 2021) or how lockdown has changed pre-professional dancers' training and life (Bruyneel et al., 2020; Man, 2020; Moorhouse, 2020; Papp-Danka and Lanszki, 2020; Tariao and Yang, 2021; Weber, 2021). Lockdown itself has meant that pre-professional training institutions alter their face-to-face dance classes to online delivery, which has been an exceptional challenge for both students and teachers as neither had any experience of such conditions (Moorhouse, 2020; Papp-Danka and Lanszki, 2020).

Although these online activities could never replace traditional dance classes, which did provide a stimulus to innovate the dance curriculum and education (Bruyneel et al., 2020). Dance teachers had to quickly design their lessons to be carried out in a much smaller space (3–5 m^2^) and often on a wooden floor or tiled floor. Accompanied with unstable internet connections, which led to low image quality, their usual detailed movement feedback was made particularly challenging (Bruyneel et al., 2020). Man (2020) and Weber (2021) noted that the change in the training environment required “new rituals” and a reflection on what could be taught safely.

Currently, there are no published data on how the lockdown due to pandemic has affected the injury profile in dancers. This study aimed to compare injury prevalence, causes, and risk factors before and after the COVID-19 lockdown in Chinese pre-professional dancers.

## Materials and Methods

This study used a cross-sectional survey design to recall injury prevalence and etiology during the two distinct periods of time: the first was a 6-month period prior to COVID-19 lockdown and the second was a 6-month period during the lockdown where participants were restricted to their homes. Ethical approval was granted by the University of Wolverhampton (07/20/YD2/UOW).

The survey used the same questionnaire that was utilized in a previous survey on injury prevalence in Chinese dancers (Dang et al., 2020); this had previously been adapted from the “Fit to Dance” Dancer and Dance Students Questionnaire (Brinson and Dick, 1996; Laws, 2005). There were 17 questions from the original questionnaire and 5 new and 5 repetitive questions to cover the “during COVID-19” period. This questionnaire focused on the student's basic information (personal data, dance education background, and dance training situation) before proceeding to their dance injury experiences in a 12-month period and during the COVID-19 period.

In the original 17 items, a few options were modified for this cohort. For instance, the options in question 1 were changed as an affiliation middle school student and a university student; for question 7, Chinese Classic Dance, Chinese Folk Dance, Chinese Dance, Sports Dance, and Dancology were added; the options of body conditioning, strength training, and fitness training were synthesized as body conditioning (question 8); the questions of hours spent every week dancing (question 8) and the hours of sleeping (question 9) were asked for the two different time periods (before and during the COVID-19); and the option of the arms/hands was subdivided into hands, elbow, wrist, and upper arm, and/or forearm (question 15).

The five new questions included dance education background (question 5 dance background and question 6 current year/grade they are into); the degree of fatigue they are feeling during the two time periods (a Likert's scale 1–10). In the last part of the questionnaire, questions focused on online classes (questions 22 and 23) and dance floors (question 24) were added for the “during COVID-19” period. Finally, five items (25, 26, 27, 28, and 29) of dance injury experience were repeated for the “during COVID-19” period. The original (Chinese) version and the translated English version of the surveys are available as Supplementary Files A, B, respectively.

### Definition of Dance Injury

Dance injury is defined as a physical problem that happens while dancing, which manifests as a pain or discomfort resulting in a modified participation, dysfunction, reduced range of movement, or the immediate cessation of dance activity. A previous study (Dang et al., 2020) highlighted the issues when participants had self-reported each injury, its site, etiology, and cause. Therefore, in this survey, we used injury severity, which was a summary of all injuries and was categorized as minor (I felt pain/uncomfortable, but it did not affect my dancing), moderate (I had to adapt my movement or could not do specific actions), moderate to severe (I had to stop dancing but no more than 1 day), and severe (I could not dance 1 day or more). Questions 15, 16, and 19 refer to the site, type, and perceived cause of injuries, respectively; respondents were asked to tick as many options as required to account for their different injuries over the defined time periods.

### Inclusion Criteria

Inclusion criteria include the dance students who are receiving full-time dance training at an university, a technical secondary school, or a vocational dance school. The dance students who have graduated or are at the master's or a higher level were omitted.

### Online Survey and Procedures

We distributed the survey using WenJuanXing (https://www.wjx.cn/), an online platform that meets the European General Data Protection Regulation (2020). We used a snowball method to contact potential respondents, initially contacting dance teachers who forwarded the link to their students and also to other dance teacher colleagues. We also requested that students forward the survey link to their peers either at the same school or in a different institution.

This online survey started on September 13, 2020 and ended on November 10, 2020. The informed consent was on the first page of the questionnaire and comprised student consent for dance students over 18 years old and additional parent consent for the dance students who were under 18. The rest of the questionnaire was only accessible once they had given consent. The Chinese school year has two holiday periods, summer (July-September) and winter (January-March). Therefore, the pre-lockdown period (6 months) corresponded to the semester from September 2019 to February 2020 (inclusive) and the lockdown period (6 months) from March to August 2020 (inclusive).

### Data and Statistical Analysis

STROBE reporting guidelines were followed (Von Elm et al., 2014). Independent variables included the two reporting periods (pre-COVID and during the lockdown), dance genre (Dancology, Chinese Dance, DanceSport, Ballet, Chinese Folk Dance, Contemporary Dance, and Chinese Classic Dance), school level (USAMS—university students and those who were also trained full time at an affiliation middle school; USHS—university students who had previously studied part-time at high school; AMS—affiliate middle school students), anthropometric data (height, body mass, and age), and sex (male and female). Dependent variables included injury prevalence, cause, site, type and severity, and the perceived cause of injury.

Differences in injury prevalence, cause, site, type and severity, and the perceived cause of injury in different periods were assessed by the Mann–Whitney *U*-test, the Kruskal–Wallis *H*-test, the McNamer test, the chi-squared test, and the Wilcoxon Signed-Ranks test. Binary Logistic Regression was used to investigate the risk factors associated with an injury event. The SPSS version 26 (SPSS, Inc., Chicago, IL, USA) was used for the aforementioned analyses, and the level for statistical significance was set at *p* ≤ 0.05.

## Results

After an initial analysis of the data, we decided to combine all Chinese dance styles (Classical Dance and Folk Dance) under the heading Chinese Dance. We removed all the weekly training hours data due to reporting inconsistencies. About 25 respondents reported injuries during the lockdown but missed answering the questions on injury incidence in the last 12-month period.

### Participants

A total of 2,111 dance students initially responded to the survey, but 25 who rejected the informed consent. The final total of 2,086 dancers [age 19 ± 2.40 years, height 168.4 ± 7.14 cm, body mass 53.2 ± 8.26 kg, and body mass index (BMI) 18.7 ± 2.1] was derived from 51 different institutions and comprised of 1,773 university students (20 ± 1.56 years, 168.5 ± 6.86 cm, 54.0 ± 7.92 kg, and BMI 18.9 ± 1.95) and 313 affiliation middle school students (15 ± 1.60 years, 167.5 ± 8.52 cm, 48.3 ± 8.48 kg, BMI 17.1 ± 2.25) while the majority of all respondents are women (*n* = 1,623, 77.8%). The main dance genres/major are Dancology (*n* = 1,183, 56.7%), Chinese Dance (*n* = 284, 13.6%), DanceSport (*n* = 214, 10.3%), Ballet (*n* = 123, 5.9%), Chinese Folk Dance (*n* = 107, 5.1%), Contemporary Dance (*n* = 81, 3.9%), and Chinese Classic Dance (*n* = 81, 3.9%). In the studied lockdown, 93% of the students (*n* = 1,938) received online classes from their schools for 3 (28.1%) to 4 months (33.6%), and they danced on the different floor types (28.22% wooden floor, 36.64% ceramic floor, 4.02% carpet, 18.01% Yoga mat, 12.07% personal dance studio floor, and 1.03% others). The complete data set is available at https://doi.org/10.6084/m9.figshare.16624207.v2.

The whole cohort (2,086 dance students) was further divided into three groups based on their dance training backgrounds and skill. The highest skilled group (USAMS) was the group at the university and the group who had also trained full time at an affiliation middle school (*n* = 409, 8 ± 2.10 years training); the next group (USHS) was university students who had previously studied part-time at high school (USHS: *n* = 1,364, 2 ± 1.44 years training); and the last group (AMS) was AMS (*n* = 313, 3 ± 1.87 years training). Age, height, body mass, and BMI significantly differ in the three groups (*p* < 0.01). The AMS cohort was significantly younger than their university counterparts (*p* < 0.05) between groups. The USAMS reported more previous years of full-time training (PYT) than the other two groups (*p* < 0.01). Generally, men reported more years prior training (PYT) than women, with a significant difference for the USAMS group (*p* < 0.01; Table 1).

**Table 1:** Respondent descriptive data: anthropometrics and dance training background.

	**Whole cohort**	**AMS**	**USAMS**	**USHS**
**Group (** ***n*** **)**	**2,086**	**313**	**409**	**1,364**
Age^†^	19.2 ± 2.40	14.9 ± 1.60 (A < UA^†^)	19.8 ± 1.71 (UA < UH^†^)	20.0 ± 1.51 (A < UH^†^)
Height^†^	168.4 ± 7.14	167.5 ± 8.52 (A < UA^†^)	169.2 ± 6.76 (UA > UH^*^)	168.3 ± 6.88 (A < UH^*^)
Body mass^†^	53.2 ± 8.26	48.3 ± 8.48 (A < UA^†^)	54.0 ± 7.85	54.0 ± 7.95 (A < UH^†^)
BMI^†^	18.7 ± 2.10	17.1 ± 2.25 (A < UA^†^)	18.8 ± 1.81	19.0 ± 1.99 (A < UH^†^)
PYT	3.2 ± 2.80	2.8 ± 1.87 (A < UA^†^)	7.7 ± 2.10 (UA > UH^†^)	1.9 ± 1.44
**Gender**	**Male**	**Female**	**Male**	**Female**	**Male**	**Female**	**Male**	**Female**
**Group (** ***n*** **)**	**463**	**1,623**	**89**	**224**	**90**	**319**	**284**	**1,080**
Age	19.1 ± 2.74	19.2 ± 2.89	14.6 ± 1.70	15.0 ± 1.55^*^	20.3 ± 1.91^†^	19.6 ± 1.63	20.1 ± 1.57	20.0 ± 1.49
Height	177.0 ± 7.56^†^	165.9 ± 4.65	173.5 ± 11.57^†^	165.1 ± 5.36	178.1 ± 5.25^†^	166.7 ± 4.72	177.8 ± 6.17^†^	165.8 ± 4.42
Body mass	62.6 ± 9.40^†^	50.5 ± 5.46	53.4 ± 11.20^†^	46.3 ± 6.06	64.4 ± 7.36^†^	51.1 ± 5.01	64.9 ± 7.43^†^	51.1 ± 5.06
BMI	19.9 ± 2.75^†^	18.3 ± 1.71	17.6 ± 2.84^*^	17.0 ± 1.94	20.3 ± 2.13^†^	18.4 ± 1.45	20.6 ± 2.51^†^	18.6 ± 1.60
PYT	3.3 ± 3.04	3.1 ± 2.72	3.1 ± 2.02	2.8 ± 1.8	8.3 ± 2.26^†^	7.5 ± 2.03	1.8 ± 1.45	1.9 ± 1.44

* *p < 0.05*.

† *p < 0.01*.

*AMS, Affiliation middle school students; USAMS, University but previously trained at affiliate school; USHS, University but previous trained at high school; BMI, Body mass index; PYT, Previous years of training (full-time)*.

### Injury Prevalence

Of 2,086 dance students, 1,145 reported injury prevalence over the entire last 12 months 54.9%, and 344 (16.5%) reported an injury during the lockdown. Injury prevalence before the lockdown was 39.6%, which was significantly dropped to 16.5% (*p* < 0.01) during the lockdown. There was no significant difference between men and women across the reporting periods (*p* > 0.05).

Compared to before the lockdown period, the injury prevalence decreased in students who are studying Dancology (*x*^2^ = 163.118, *p* < 0.01), Contemporary Dance (*x*^2^ = 11.500, *p* < 0.01), Chinese Dance (*x*^2^ = 11.658, *p* < 0.01), and DanceSport (*x*^2^ = 32.764, *p* < 0.01) during the lockdown. Furthermore, Chinese dancers had the highest prevalence (23.5%) during the lockdown (*x*^2^ = 33.962, *p* < 0.01) while before the lockdown, the highest injury prevalence (43.3%) was reported in Dancology students (*x*^2^ = 23.252, *p* < 0.01).

Compared to the university level groups (*p* < 0.01), the AMS group reported a higher injury prevalence (30.4%) during the lockdown but had a lower prevalence (26.2%) before the lockdown (*p* < 0.01). For Chinese Dance, the AMS group had a significantly higher prevalence (36.5%) than the USHS group during the lockdown (*x*^2^ = 23.375, *p* < 0.01), but the former group reported a lower (27%) prevalence than the latter group (*x*^2^ = 7.137, *p* < 0.05) before the lockdown. For those who are studying Dancology, the AMS group reported a higher injury prevalence (41.7%) than the other two groups during the lockdown (*x*^2^ = 21.430, *p* < 0.01) (Table 2).

**Table 2:** Dance injury prevalence in two different periods (%).

**Genres**	**Backgrounds**	**Before lockdown**		**During lockdown**	
Dancology (*n* = 1,183)	AMS	27.8%	43.3%^†^ (Be > Du)^†^	41.7%^†^	14.9%
	USAMS	45%		15.6%	
	USHS	43.5%		13.6%	
Ballet (*n* = 123)	AMS	16.5%	25.2%	22.7%	20.3%
	USAMS	60%^†^		12%	
Contemporary dance (*n* = 81)	AMS	80%	43.2% (Be > Du)^†^	20%	13.5%
	USAMS	47.8%		8.7%	
	USHS	37.7%		15.1%	
Chinese dance (*n* = 451)	AMS	27.0%	36.1% (Be > Du)^†^	36.5%^†^	23.5%^†^
	USAMS	40.6%		24.2%^†^	
	USHS	39.8%^*^		13.4%	
DanceSport (*n* = 214)	AMS	41.7%	33.6% (Be > Du)^†^	8.3%	7.9%
	USAMS	35.9%		5.7%	
	USHS	32.2%		8.7%	
Others (*n* = 34)	AMS	38.5%	38.2%	23.1%	26.5%
	USAMS	37.5%		37.5%	
Total (*n* = 2,086)	AMS	26.2%	39.6%^†^	30.4%^†^	16.5%
	USAMS	43.5%^†^		16.4%	
	USHS	41.5%^†^		13.3%	

*“Chinese Dance” included Chinese Classic Dance and Chinese Folk Dance*;

* *p < 0.05*;

† *p < 0.01*.

*AMS: Affiliation middle school students; USAMS: University but previous trained at affiliate school; USHS: University but previous trained at high school; Be: Before lockdown; Du: During lockdown*.

### Injury Severity

In the two sub-periods, students reported a higher prevalence of minor and moderate injuries than moderate–severe and severe injuries (*p* < 0.01). However, compared to before the lockdown period, the prevalence of moderate-to-severe injuries increased by 4.1% (*x*^2^ = 4.019, *p* < 0.05) during the lockdown period. The USAMS group reported more moderate-to-severe injuries before the lockdown (*x*^2^ = 6.863, *p* < 0.01) than the USHS group but no significant difference during the lockdown. Approximately 14.5% of all respondents reported that their injuries resulted in days off from dancing; days lost to severe injury were similar among the three groups in the two periods (Table 3).

**Table 3:** Injury severity in two periods (%).

**Severity**	**Backgrounds**	**Before lockdown**		**During lockdown**	
Minor^†^	AMS	39.0%	46.5%	39.0%	43.4%
	USAMS	42.7%		40.3%	
	USHS	48.8%		46.7%	
Moderate^†^	AMS	50%	47.8%	48.4%	45.6%
	USAMS	55.1%		50.8%	
	USHS	45.2%		42.3%	
Moderate to severe	AMS	9.8%	10.4%	12.6%	14.5%
	USAMS	15.7%^†^	(Be < Du)^*^	16.4%	(MS > S)^*^
	USHS	8.8%		14.8%	
Severe	AMS	13.4%	9.9%	12.6%	9.0%
	USAMS	10.7%		10.5%	
	USHS	9.2%		6.6%	
Days lost	Total groups	46.8 ± 63.23	34.8 ± 39.83

*Mo and Mi, Moderate and minor injuries; Be and Du, Before and during lockdown; MS and S, Moderate to severe and severe injuries*.

* *p < 0.05*.

† *p < 0.01*.

### Tissue Injury and Sites

Respondents reported a higher prevalence of muscle and joint/ligament injuries than bone and tendon injuries (*p* < 0.01) in the two periods. The prevalence of muscle injuries was higher than that of joint/ligament injuries before the lockdown (*p* < 0.01), but the prevalence of both was the same during the lockdown (47 vs. 46.5%). Muscle injuries decreased by 15% during the lockdown compared to before the lockdown (*U* = 127,797, *z* = −3.177, *p* < 0.01) (Table 4).

**Table 4:** Tissue injury in two periods (%).

**Tissue**	**Backgrounds**	**Before lockdown**		**During lockdown**	
Muscle^†^	AMS	45.1%	61.5%	48.4%	47.0%
	USAMS	61.8%^†^	(M > J)^†^	47.8%	(Be > Du)^†^
	USHS	63.8%^†^		54.4%	
Bone	AMS	17.1%	17.2%	14.7%	16.0%
	USAMS	22.5%		23.9%	
	USHS	15.6%		13.7%	
Joint/ligament^†^	AMS	51.2%	50.7%	50.5%	46.5%
	USAMS	46.6%		43.3%	
	USHS	51.9		45.6%	
Tendon	AMS	15.9%	11.9%	6.3%	11.1%
	USAMS	12.4%		17.9%	
	USHS	11.1%		11.0%	
Other	AMS	11.0%	7.0%	7.4%	5.8%
	USAMS	9.0%		6.0%	
	USHS	5.8%		5.0%	

† *p < 0.01*.

*AMS, Affiliation Middle School students; USAMS, University but previous trained at affiliate school; USHS, University but previous trained at high school; M, Muscle injury, J, Joint/Ligament injury; Be, Before lockdown; Du, During lockdown*.

The top six reported injury sites were the lower back, knees, feet, ankles, shoulders, and groin/hip-joint in the two sub-periods. During the lockdown, the injuries on the lower back (*p* < 0.01), feet (*p* < 0.01), and shoulders (*p* < 0.01) decreased significantly compared to before the lockdown period. However, knees, ankles, and groin/hip did not vary significantly despite an overall drop in injury prevalence. The USAMS group reported a significantly high prevalence on the lower back (*p* < 0.05) than the other two groups while for the AMS group, it was the groin/hip-joint injuries (*p* < 0.05) in the two periods (Table 5).

**Table 5:** Injury sites in two periods (%).

		**Arms**	**Hands**	**Elbow**	**Wrist**	**Shoulders**	**Neck**	**U back**	**L back**	**Ribs**	**Pelvis**	**Groin/Hip**	**U legs**	**Knees**	**L legs**	**Ankles**	**Feet**
Before lockdown	C	8.8	6.2	7.4	9.4	16.1	10.7	7.0	45.4	1.7	5.3	14.4	15.9	32.5	7.5	24.0	30.4
	A	6.1	6.1	4.9	7.3	7.3	6.1	6.1	37.8	1.2	6.1	28.1^†^	8.5	23.2	6.1	28.2	22.0
	UA	6.2	4.5	5.1	9.6	14.6	14.0	6.3	53.4^*^	1.7	3.4	14.0	9.0	34.8	5.1	19.1	29.2
	UH	10.1	6.7	8.5	9.7	17.8^*^	10.3	7.4	44.0	1.8	5.8	12.5	19.1^†^	33.0	8.5	24.9	32.0
During lockdown	C	12.5	7.0	8.1	6.7	7.3^†^	7.3	4.7	29.1^†^	1.2	2.6^*^	10.5	8.4^†^	29.9	5.5	23.6	18.9^†^
	A	6.3	4.2	3.2	6.3	6.3	2.1	5.3	29.5	0.0	4.2	20.0^†^	5.3	28.4	0.0	15.8	14.7
	UA	14.9	6.0	7.5	4.5	6.0	10.5^*^	9.0^*^	38.8^*^	1.5	1.5	7.5	10.5	29.9	6.0^*^	26.9	22.4
	UH	14.8^*^	8.8	10.9^*^	7.7	8.2	8.8	2.8	25.3	1.7	2.2	6.6	9.3	30.8	8.2^†^	26.4^*^	19.8

* *p < 0.05*.

† *p < 0.01*.

*C, whole cohort; AMS, Affiliation Middle School students; USAMS, University but previous trained at affiliate school; USHS, University but previous trained at high school*.

### Injury Causes

During the lockdown, the recurrence of old injury (37.2%), fatigue (33%), and insufficient warm-up (26.5%) were still the top perceived causes while respondents reported a significant increase of an unsuitable floor (*p* < 0.01), a cold environment (*p* < 0.05), and set/props (*p* < 0.05) as the causes for their injury. Between-group analysis highlighted that the USAMS group reported that their causes were the recurrence of an old injury in the two sub-periods (*p* < 0.05). During the lockdown, the AMS group reported an increase in “accident” as being the cause of injury (*p* < 0.05; Table 6).

**Table 6:** Injury causes in two periods (%).

**Different periods**	**Different training backgrounds**	**Fatigue**	**Limited/bad flexibility**	**Unsuitable floor**	**Cold environment**	**Insufficient warm up**	**Insufficient cooldown**	**New/difficult choreography**	**Repetitive movement**	**Partnering work**	**Incorrect technique/training**	**Ignoring early warning signs**	**Lack the sense of self-protection**	**Recurrence of old injury**	**Inadequate diet/hydration**	**Set/props**	**Costume/shoes**	**Rehearsal schedule**	**Accident**	**Others**
Before lockdown	C	36.1	24.2	9.9	5.7	29.1	10.1	8.5	10.4^*^	1.9	22.5^†^	13.0	28.5^†^	40.1	2.9	2.3	3.2	3.9	19.3	1.1
	AMS	37.8	28.0^†^	11.0	2.4	28.0	9.8	2.4	4.9	0.0	23.2	12.2	28.0	28.0	1.2	0.0	3.7	2.4	17.1	4.9
	UAMS	43.8^*^	12.4	9.6	8.4	19.7	9.0	14.6^†^	12.9^*^	3.4	19.7	14.6	25.8	53.9^†^	3.4	1.7	4.5	3.9	18.0	1.1
	USHS	33.4	27.4^†^	9.9	5.3	32.2^†^	10.4	7.6	10.4	1.8	23.3	12.5	29.3	37.5	3.0	2.8	2.7	4.1	20.0	0.5
During lockdown	C	33.0	19.2	31.1^†^	9.3^*^	26.5	12.8	7.6	6.7	0.6	15.7	10.2	20.1	37.2	2.0	4.7^*^	1.2	2.6	16.9	0.3
	AMS	37.9	16.8	25.3	5.3	27.4	11.6	6.3	7.4	0.0	14.7	6.3	21.1	37.9	3.2	3.2	1.1	2.1	22.1^*^	1.1^†^
	UAMS	35.8	14.9	29.9	6.0	22.4	7.5	11.9	3.0	3.0^*^	11.9	10.4	14.9	55.2^*^	3.0	3.0	1.5	4.5	25.4^*^	0.0
	USHS	29.7	22.0	34.6	12.6	27.5	15.4	6.6	7.7	0.0	17.6	12.1	21.4	30.2	1.1	6.0	1.1	2.2	11.0	0.0

*AMS, Affiliation middle school students; USMAS, University but previous trained at affiliate school; USHS, University but previous trained at high school; C, Whole cohort*.

* *p < 0.05*.

† *p < 0.01*.

### Injury Risk Factors

The developed binary logistic model had an overall prediction accuracy of 85.9% before the lockdown and 87.2% during the lockdown. There was no significant difference between the predicted results and actual survey results (*p* > 0.05), indicating that the models have a strong efficacy.

The Wilcoxon Signed Ranks test indicated that the student fatigue (*Z* = −11.145, *p* < 0.01) decreases (from 5 ± 2.36 to 4.43 ± 2.29) but the hours of sleep increase (from 7.3 ± 1.57 to 7.9 ± 1.82) significantly (*Z* = −16.869, *p* < 0.01) during the lockdown than the period before (*p* < 0.01). Binary Logistic Regression analysis showed that fatigue was a negative risk factor of injury during the lockdown (*B* = 0.173, *p* < 0.01), every degree increases in fatigue approximately increase the risk of injury prevalence by 1.189 times (OR = 1.189, 95% CI: 1.114–1.270). Furthermore, the reported number of hours of sleeping on a night had a significantly positive influence (*B* = −0.116, *p* < 0.01) on injury prevalence with every extra hour of sleep potentially decreasing the injury by 0.89 times (OR = 0.890, 95% CI: 0.818–0.968) during the lockdown. Furthermore, the AMS group reported no significant fatigue difference (*Z* = −1.861, *p* > 0.05), but the other two groups reported a lower fatigue degree during the lockdown period (USAMS: *Z* = −8.168, *p* < 0.01; USHS: *Z* = −8.159, *p* < 0.01).

Respondents indicated that if they suspect an injury, they will take pain killers (10.6%) and continue to dance but carefully (43.7%). Binary Logistic Regression analysis indicated that behaviors, such as taking pain killers (*B* = 0.596, *p* < 0.05) and continuing to dance, however carefully (*B* = 0.589, *p* < 0.001) can approximately increase injury prevalence almost two times (OR = 1.814, 95% CI: 1.145–2.874 and OR = 1.801, 95% CI: 1.308–2.480, respectively) in the lockdown period.

Most of the Chinese dance students (70.7%) reported that their dance teachers made time available for a cooldown after dancing. Before the lockdown, timing being set aside for a cooldown by teachers was shown to have a significant positive influence (*B* = −0.349, *p* < 0.05) on injury prevalence, which could decrease by 0.705 times (OR = 0.705, 95% CI: 0.509–0.978). At the same period, age was a negative factor for injury prevalence (*B* = 0.234, *p* < 0.01), with each year older increasing the risk of injury by 1.263 times (OR = 1.263, 95% CI: 1.168–1.366).

## Discussion

The purpose of this study was to examine injury prevalence, causes, and risk factors in Chinese pre-professional dancers before and during the COVID-19 lockdown. To our knowledge, this study is the largest dance injury survey in terms of participant numbers, which has replicated the established methodologies (i.e., questionnaire), thereby allowing appraisals with available data (Laws and Apps, 2005; Liederbach and Richardson, 2007; Dang et al., 2020; International Olympic Committee Injury et al., 2020). For instance, contrary to a previous study on Chinese dancers which revealed that men demonstrate a higher injury prevalence than women (Dang et al., 2020), which was not replicated in the current survey. It is noteworthy that this lack of sex difference in injury prevalence in pre-professional dancers has been previously confirmed (Lee et al., 2017).

The main finding was that compared to before the lockdown, injury prevalence significantly dropped from 39.6 to 16.5% (*p* < 0.01) during the lockdown. The binary logistic model used for the purposes of this study highlighted a positive benefit of decreased feelings of fatigue and longer hours of sleep on the reduced injury prevalence during the lockdown period. Fatigue has been repeatedly reported as the main injury cause (Brinson and Dick, 1996; Laws and Apps, 2005; Angioi et al., 2009; Twitchett et al., 2010; Day et al., 2011; Dang et al., 2020; Kozai et al., 2020; Tjukov et al., 2020). Getting adequate sleep has previously been linked to a decreased fatigue degree with a concomitant decrease in injury prevalence (Mainwaring and Finney, 2017). Teachers reported that the hours spent on dance training did not change during the lockdown period, but the intensity of the class was limited because students did not have the space to “travel” while dancing (the allegro section of a dance class). Thus, the reduced feelings of fatigue and longer sleep hours could explain why injury prevalence dropped significantly during the lockdown.

A lower fatigue degree of the university groups (USAMS and USHS) (*p* < 0.01) made a positive contribution to a drop in injury prevalence during the lockdown. The AMS cohort, on the other hand, reported a consistent feeling of fatigue (*p* > 0.05), and a higher percentage of accidents (*p* < 0.05) across the two lockdown periods could explain no decrease in its injury prevalence. Meanwhile, the other two older groups reported reduced feelings of fatigue, the younger AMS group possibly had less effective coping strategies, and therefore the lockdown period affected them more (Pickard and Risner, 2020).

This survey is the first to report injury prevalence in Dancology major (*n* = 1,183), which is a very important major in pre-professional Chinese dance training. The Dancology and Chinese Dance students reported the highest injury prevalence in the current survey, the latter genre's injury prevalence also increased by 17% (before the lockdown) to 30% (during the lockdown) since the 2018 survey (Dang et al., 2020). These two majors had a significant decrease in injury prevalence between before and during the lockdown period. This could be due to the diversity of their training background, both previous full- and part-time training and often a limited number of years of previous training. The injury data from the ballet genre cohort supports this concept as almost all ballet students came from a full-time ballet training background and had the longest years of previous training; in this cohort, there was a little difference in injury prevalence between before and during the lockdown period. The years of previous full-time training could be a possible injury factor, which needs further analysis and study.

The present data also revealed a decreased prevalence of minor and moderate injuries but a significant increase in moderate-to-severe injuries during the lockdown. Thus, the present finding could be linked to dance students' physical deconditioning due to the experience of the reduced training load during the lockdown (Bruyneel et al., 2020). Indeed, Angioi et al. (2009) indicated that increased severity of injuries has been often associated with a reduced level of lower body muscular power.

In line with previous studies (Hincapié et al., 2008; Magida, 2009; Malkogeorgos et al., 2011; Dang et al., 2020), we found that muscle and joint/ligament injuries remained to be the main type of injuries and did not change in the two sub-periods (i.e., before and during the lockdown). The main injury sites of the knees, ankles, and groin/hip-joint also remained unchanged in the two studied periods, but injuries on the lower back, feet, and shoulders decreased significantly in the lockdown period. There was no obvious reason for an injury decline in these sites given that the “reoccurrence of an old injury” did remain the main perceived cause, whereas “unsuitable floor” as a possible cause of injury increased in the lockdown period. Nevertheless, this finding contradicts with what might be expected, which would be a positive association between an unsuitable floor and injuries on the lower limbs and back (Pratt, 1989; Ferber et al., 2009). Previous research on dance floors has reported that the modifiability/mechanical properties of the dance floors (construction of different stages and class floors) greatly influence injury rates (Pappas et al., 2012; Wanke et al., 2012; Hopper et al., 2014a,b; Hackney et al., 2015) though no study has examined the effect of dancing at home on tiles or concrete.

The fact that ~14.5% of all respondents reported that their injuries resulted in days off from dancing, supporting the notion that using a time-loss definition for injury could underestimate injury burden (Kenny et al., 2018) and may not be suitable for determining injury prevalence (Swain et al., 2019). Thus, we recommend that scientists should find an appropriate methodology to try to collect authentic data from the surveys that recognize a time-off training, modified-movement training, or reduced training due to an injury.

It is reasonable to assume that the present results may have been influenced by methodological limitations. For example, although respondents were asked to report the number of hours of training in a week before and during the lockdown, the data were inconsistent and therefore omitted. However, 25 dance students only reported their injuries during the lockdown period but missed to answer the question that “whether you had an injury in the last 12 months?”

Within the limitation of this study, it was concluded that, although injury prevalence dropped significantly during the first COVID-19 lockdown in Chinese dance students, the main injury characteristics remained the same. Decreased fatigue and longer hours of sleep could explain the aforementioned drop in injury prevalence during the lockdown. However, injury severity increased possibly due to deconditioning and reduced training load experienced during the lockdown. The main injury sites such as the knees, ankles, and groin/hip-joint also remained unchanged in the two studied periods, but the lower back, feet, and shoulders decreased significantly in the lockdown period. Because of different training environments, the unsuitable floor (tiles or concrete) increased significantly as a main perceived cause of injury; but the recurrence of old injury and fatigue remained as the top two causes during the lockdown. Further studies are needed to investigate how dance training changes affect injuries to offer an effective intervention.

## Data Availability Statement

The raw data supporting the conclusions of this article will be made available by the authors, without undue reservation. The complete data set can be found at https://doi.org/10.6084/m9.figshare.16624207.v3.

## Ethics Statement

The studies involving human participants were reviewed and approved by School of Sport Ethics Committee, University of Wolverhamtpon, UK (07/20/YD2/UOW). Written informed consent to participate in this study was provided by the participants, or where necessary, the participants' legal guardian/next of kin.

## Author Contributions

YD: adaptation of survey, translation of survey, data collection, data analysis, and writing of the article. YK and RC: writing of the article. MW: adaptation of survey, data analysis, and writing of the article. All authors contributed to the article and approved the submitted version.

## Funding

This work was supported by the China Scholarship Council for their financial contribution (YD).

## Conflict of Interest

The authors declare that the research was conducted in the absence of any commercial or financial relationships that could be construed as a potential conflict of interest.

## Publisher's Note

All claims expressed in this article are solely those of the authors and do not necessarily represent those of their affiliated organizations, or those of the publisher, the editors and the reviewers. Any product that may be evaluated in this article, or claim that may be made by its manufacturer, is not guaranteed or endorsed by the publisher.

## Supplementary Material

Supplementary File A Survey in Chinese.

Supplementary File B Survey in English.
