# Supplementary material for: Prevalence and Risk Factors of Dance Injury During COVID-19: A Cross-Sectional Study From University Students in China
Source: Front Psychol. 2021 Oct 27;12:759413. doi: 10.3389/fpsyg.2021.759413 (PMC8579743; doi:10.3389/fpsyg.2021.759413)
Supplement: Supplementary File A — Survey in Chinese. [file Data_Sheet_1.pdf]

# 舞蹈专业学生损伤调查问卷 (改编自 Fit to Dance 2)

知情同意：

请仔细阅读下列声明。阅读完后请你点击你是否愿意接受这则声明且很高兴将您的答案用于此研究目的。

- 第一份调查重点是在过去 12 个月里，发生在舞蹈中的损伤。你将被要求填写个人信息，包括你的年龄、身高和体重，你在哪里训练，你每天/周跳舞几个小时等问题。此问卷 15 分钟内即可完成。
- 我知道我的信息将被完全地保密，并且每个人只会有一个唯一的标识码（下一页）。
- 我知道我没有义务完成这项调查，并且我可以在任何时候出于任何原因停止调查。
- 调查结果是保密的，且将只会与研究团队内的其他人讨论交流。

你已满 18 岁▢，你未满 18 岁▢。

你接受这些声明，请点击此处▢ (开始填写问卷)

如果你不接受这些声明，请点击此处▢(结束，至感谢页面)

未满 18 岁，你接受这些声明，请点击此处▢ (下一项)

如你未满 18 岁，则此声明也需父母同意。征得父母同意后，请点击此处▢(开始填写问卷)

如果你/父母不接受这些声明，请点击此处▢(结束，至感谢页面)

日期_____年_____月_____日

## 个人标识码与个人信息

1. 你目前是（单选）？

   a 舞蹈专业附中生

   b 舞蹈专业本科生

2. 性别______；

   年龄______(yrs);

   体重______(kg);

   身高______(cm). (单选)

3. 你就读于哪所学校？（单选）

   a 北京舞蹈学院

   b 沈阳音乐学院

   c 上海电影艺术学院

   d 四川音乐学院

   e 天津体育学院

   f 燕山大学

   g 其它，请填写________________________________

4. 你进入全日制舞蹈训练时是几岁？____(如你是/曾是“附中生”，则填写你进入附中时的年龄；如您是“普高生”，则填写你进入本科时的年龄。单选)

5. 进入本科前，你的专业学习背景是？ （普高生，舞蹈专业附中生）

6. 你目前是几年级的学生? (单选)

   a 附中 1 年级

   b 附中 2 年级

   c 附中 3 年级

   d 附中 4 年级

   e 附中 5 年级

   f 附中 6 年级

   g 附中 7 年级

   h 本科 1 年级

   i 本科 2 年级

   j 本科 3 年级

   k 本科 4 年级

l　其他，请补充——————

7. 你的主要舞种/专业是什么？(请注意分清 c、d、e 和 g 选项。单选)

a　舞蹈学

b　芭蕾舞

c　现代舞/现代舞编导

d　中国民族民间舞

e　中国古典舞

f　中国舞

g　国际标准舞

h　其它，请填写________________________________

8. 在学校关闭之前（疫情发生前，2020 年 2 月之前），你平均每周花费几个小时在下列选项中？(请填写下列每个选项，如果有个别项目没做，请选择“0”)。

a　专业课　____________小时

b　排练　____________小时

c　表演　____________小时

d　身体素质训练（普拉提、跑步、健身操等）　____________小时

e　其它，请填写______________，__________小时

学校关闭后（疫情期间，2020 年 2 月之后），你平均每周花费几个小时在下列选项中？(请填写下列每个选项，如果有个别项目没做，请选择“0”)。

a　专业课　____________小时

b　排练　____________小时

c　表演　____________小时

d　身体素质训练（普拉提、跑步、健身操等）　____________小时

e　其它，请填写______________，__________小时

9. 你平均每晚睡几个小时? (单选)

a　疫情前____小时

b　疫情期间____小时

10. 疫情前，你的疲劳程度为_____；

疫情时，你的疲劳程度为_____ (1-10, 1 代表不疲劳, 10 代表非常疲劳）(单选)

11. 你是否做热身活动？(单选)

a 是

b 否

11.1 如果“是”, 多久? (如果你在下列选项之前做了热身活动，请选择符合你情况的时长，如果在个别选项之前不做热身活动，请选择“0”) (单选)

| | 0 | 1-5 | 6-10 | 11-15 | around 20 |
|---|---|---|---|---|---|
| a 课前 | ___ | ___ | ___ | ____ | ____ |
| b 排练前 | ___ | ___ | ___ | ____ | ____ |
| c 演出前 | ___ | ___ | ___ | ____ | ____ |

12. 你是否做放松活动？(单选)

a 是

b 否

12.1 如果是，多久？(如果你在下列选项之后做了放松活动，请选择符合你情况的时长，如果在个别选项之后不做放松活动，请选择“0“) (单选)

| | 0 | 1- 5 | 6-10 | 11-15 | around 20 |
|---|---|---|---|---|---|
| a 课后 | ___ | ___ | ___ | ____ | ___ |
| b 排练后 | ___ | ___ | ___ | ____ | ___ |
| c 演出后 | ___ | ___ | ___ | ____ | ___ |

13. 学校为热身活动预留时间了吗？ 是 ____ 否 ____ (单选)

学校为放松活动预留时间了吗？是 ____否 ____ (单选)

## 损伤信息

此研究中的舞蹈损伤被定义为发生在舞蹈中的身体问题，表现为疼痛或不适，并导致舞者：作出相对应的调整动作以便继续跳舞、功能障碍、活动幅度减小、甚至导致立即停止任何舞蹈活动。

14. 在过去的 12 个月里，你是否有损伤？（包括长期损伤，虽发生在 12 个月以前，但近 12 个月内仍持续影响你跳舞） (单选)

    是_____ 否 _____

15. 如果你在过去 12 个月里有损伤，你损伤发生的部位在哪些部位？ (多选题)

    a ☐胳膊（大臣和小臂）_____
    b ☐手______
    c ☐肘关节_____
    d ☐腕关节___
    e ☐肩关节______
    f ☐颈部______
    g ☐上背部（胸椎）______
    h ☐下背部（腰椎）______
    i ☐肋骨/胸廓______
    j ☐骨盆______
    k ☐髋关节______
    l ☐大腿 ______
    m ☐膝关节______
    n ☐小腿______
    o ☐踝关节______
    p ☐足______

16. 在过去的 12 个月里，在训练、排练和表演中你是否有下列损伤？ (多选题)

    a ☐肌肉损伤
    b ☐骨损伤
    c ☐关节/韧带损伤
    d ☐腱损伤（肌腱或跟腱）

e 其它损伤，请补充______

17. 你的损伤严重程度是？ (多选题)

a 轻度(我仍可以跳舞，不受影响)

b 中度(我必须在动作上作出适当的调整或有一些特定的动作做不了)

c 严重(至少有 24 小时不能跳舞)

如果严重，共多少天？ _____天(单选)

18. 因为你的损伤，你寻求过哪些专业的帮助？ (多选题)

a) 理疗师
b) 医生
c) 舞蹈/运动科学领域的专家/咨询师
d) 正骨/整骨
e) 整脊师
f) 按摩
g) 针灸
h) 营养学家
i) 心理学家
j) 普拉提
k) 没有做任何措施
l) 其它，请补充______

19. 在过去 12 个月里，你认为你的损伤原因是什么？ (多选题)

a) 疲劳
b) 有限制的软度/柔韧性差
c) 不合适的地板
d) 环境温度低
e) 不充足/不适当的热身活动
f) 不充足/不适当的放松活动
g) 新的/难度大的编舞
h) 不同的剧目
i) 重复的动作
j) 搭档合作
k) 不正确的技巧/训练
l) 忽视早期警告信号
m) 缺乏自我保护意识

n) 旧伤复发

o) 饮食/补水不足

p) 舞台场景/道具

q) 服装/鞋

r) 不合适的排练计划

s) 其它，请补充___________________

20. 如果你猜想自己有损伤，你会怎么做？(多选)

a 寻找专业的医学治疗

b 告诉其他人

c 自己采取预防措施

d 吃止痛药

e 继续跳舞，但小心点儿

f 忽视它

g 隐藏它

h 其它，请补充__________________

21. 谁是指导你返回舞蹈训练过程中最影响你的人？(多选题)

a 专业医疗

b 老师

c 你自己

d 其它，请补充____________

22. 在本次疫情期间（2020 年 2 月份-至今）你是否在上过网络课程？ 是 ____ 否 ____ (单选)

23. 如果“是”，网课持续多久了？(单选题)

a 1 个月

b 2 个月

c 3 个月

d 4 个月

e 5 个月

f 6 个月

g 超个 6 个月

24. 疫情隔离在家期间，你在_______上跳舞？

a 家用木地板

b 瓷砖

c　地毯

d　瑜伽垫

e　个人舞蹈室地板（地胶）

f　其它，请补充____________

25. 在此次疫情隔离在家期间，你是否有舞蹈损伤的发生？是 _ 否_? (单选题)

26. 发生在哪些部位? (“有 “损伤)

a　胳膊（大臂和小臂）_____

b　手______

c　□肘关节_____

d　□腕关节___

e　□肩关节______

f　□颈部______

g　□上背部（胸椎）______

h　下背部（腰椎）______

i　肋骨/胸廓______

j　骨盆______

k　□髋关节______

l　□大腿 ______

m　□膝关节______

n　□小腿______

o　□踝关节______

p　□足______

27. 在过去的 12 个月里，在训练、排练和表演中你是否有下列损伤？ (多选题)

a　□肌肉损伤

b　□骨损伤

c　□关节/韧带损伤

d　□腱损伤（肌腱或跟腱）

e　其它损伤，请补充______

28. 如果“有损伤”，你的损伤严重程度属于？(多选题)

a　轻度 (我仍可以跳舞，不受影响)

b　中度(我必须在动作上作出适当的调整或有一些特定的动作做不了)

c　重度(至少有 24 小时不能跳舞)

如果是严重，多久？___天(单选)

29. 在过去 12 个月里，你认为你的损伤原因是什么？ (多选题)

a) 疲劳
b) 有限制的软度/柔韧性差
c) 不合适的地板
d) 环境温度低
e) 不充足/不适当的热身活动
f) 不充足/不适当的放松活动
g) 新的/难度大的编舞
h) 不同的剧目
i) 重复的动作
j) 搭档合作
k) 不正确的技巧/训练
l) 忽视早期警告信号
m) 缺乏自我保护意识
n) 旧伤复发
o) 饮食/补水不足
p) 舞台场景/道具
q) 服装/鞋
r) 不合适的排练计划
s) 其它，请补充__________________

## 调查结束

## 非常感谢你花费时间完成这项调查
