# Supplementary material for: Prevalence and Risk Factors of Dance Injury During COVID-19: A Cross-Sectional Study From University Students in China
Source: Front Psychol. 2021 Oct 27;12:759413. doi: 10.3389/fpsyg.2021.759413 (PMC8579743; doi:10.3389/fpsyg.2021.759413)
Supplement: Supplementary File B — Survey in English. [file Data_Sheet_2.PDF]

Dance Students Questionnaire

Informed consent:

Please read the following statements carefully. At the end you will be able to click whether you accept this statement and are happy for your answers to be used for research purposes.

- This first survey focuses on injuries that you have had whilst dancing over the last 12 months. You will also be asked to complete personal information that included your age, height and weight, where you train, how many hours you dance a day/week. It should take only 15 minutes to complete.

- I know my information will be totally confidential and I will only be recognised by a unique identifier code (next page).

- I know that I am not obliged to complete the survey and that I can stop the survey at any point and for any reason.

- The survey results are confidential and will only be communicated to others within the research team.

Please click here if you accept these statements ☐ (continues to next page)
Please click here if you do not accept these statement ☐ (continues to a thank you page)
Date ....

Identifier Code and Personal Information

1. Are you currently? (single choice)

   a Affiliation middle school students

   b University dance students

2. Gender______； Age______(yrs); Weight______(kg); Height______(cm). (single choice question)

3. What is your school's name? (single choice)

   a Beijing Dance Academy

   b Shenyang Music Academy

   c Shanghai Movie Art Academy

   d Sichuan Music Academy

   e Nanjing Art Academy

   f Yanshan University

   g Middle School Affiliated to Hainan Song and Dance Theatre

   h Others, please specify________________________________

4. What the age when you entered full-time training? ____ (single choice)

5. What is your dance education background?

   a High school students

   b Affiliation middle school students

6. What grade are you currently in? (single choice)

   a Grade 1 Affiliated Middle School

   b Grade 2 Affiliated Middle School

   c Grade 3 Affiliated Middle School

   d Grade 4 Affiliated Middle School

   e Grade 5 Affiliated Middle School

   f Grade 6 Affiliated Middle School

   g Grade 7 Affiliated Middle School

   h 1st Year University

   i 2nd Year university

   j 3rd Year university

   k 4th Grade University

   l Other, please specify________________________________

7. If you are in affiliation middle school. What is your main dance form/major? (sin...)
   - a Chinese Dance (contain Chinese folk dance and Chinese classical dance)
   - b Ballet
   - c Contemporary Dance (maybe need to be updated)
   - d Sports Dance
   - e Other, please specify________________________________

8. How many hours on average do you do a week of before your school shut down? (fill in every option, if you don't do one/some of them, just choice "0" option.)
   - a Classes number of hours____________
   - b Rehearsal number of hours ____________
   - c Performing number of hours ____________
   - d Body conditioning number of hours ____________
   - e Other, please specify______________ number of hours __________

   How many hours on average do you do a week of after your school shut down? (fill in every option, if you don't do one/some of them, just choice "0" option.)
   - f Classes number of hours____________
   - g Rehearsal number of hours ____________
   - h Performing number of hours ____________
   - i Body conditioning number of hours ____________
   - j Other, please specify______________ number of hours __________

9. How many hours of sleep a night on average do you…? (single choice in each option)
   - a Before COVID-19 ___
   - b During COVID-19 ___

10. Hoe fatigued did you feel feel? (0-10, 0 means no fatigue, 10 means very fatigue）
   - a Before COVID-19 ___
   - b During COVID-19 ___

11. Do you warm up… (ie at least 10 minutes of pulse raising activity, joint mobilization, and short stretches) (single choice)
   - a Yes
   - b No

If yes, for how long? (ie if you only don't warm up before class or rehearsal or performance, just chose "no" for it or them) (single choice in each option)

		0	1-5	6-10	11-15	around 20
a	before class	___	___	___	____	____
b	before rehearsal	___	___	___	____	____
c	before performance.	___	___	___	____	____

12. Do you cool down… (ie at least 10 minutes of pulse lowering, re-mobilization and stretching) (single choice)

    a Yes

    b No

If yes, for how long? (ie if you only do warm up before class, please chose no for rehearsal or performance) (single choice in each option)

		0	1- 5	6-10	11-15	around 20
d	before class	___	___	___	____	___
e	before rehearsal	___	___	___	____	___
f	before performance.	___	___	___	____	___

13. Is time set aside in your school to Warm up? Yes ____ no ____ (single choice)
    Is time set aside in your school to Cool down? Yes ____no ____ (single choice)

Injury Information

Dance Injury is defined as a physical problem that happened whilst dancing, which represents/shows pain or discomfort and results in modified participation, dysfunction, reduced range of movement and even stopping you immediately in any dance activity

14. In the last 12 months (include long-term injury over last 12 months but affected you in last 12 months), did you have dance injury(ies)? (single choice)
    Yes_____ no _____
15. If you did have injuries in the last 12 months, where were the sites of injury? (multiple choice)
    a Upper arm/forearm
    b hands
    c Elbows
    d Wrists
    e shoulders
    f neck
    g upper back
    h Lower back
    i Ribs
    j Pelvis
    k Hip joints
    l Thighs
    m Knees
    n Lower legs
    o Ankles
    p Feet
16. Have you had any of the following injuries in training, rehearsal and/or performance in the last 12 months? (multiple choice)
    a Muscle number__
    b Bone number__
    c joint/ligament number__
    d tendon number__

e other, please define ______ number__

17. How severity was of your injury? (multiple choice)

a Minor (I can still dance fully)

b Moderate (I have to adapt my movement or can't do certain movements)

c Severe (can't dance for at least 24 hours)

If severe, how many days can take those ones? ___days (single choice)

18. What type of professional help did you initially have for your injuries? (multiple choice)

a) Physiotherapist
b) general practitioner
c) specialist/consultant
d) osteopath
e) chiropractor
f) Masseur
g) Acupuncturist
h) Dietician
i) Counsellor
j) Psychologist
k) Pilates
l) Did nothing
m) Other, please specify______

19. What do you think was the cause of these injuries in the last 12 months? (multip)

a) Fatigue
b) Limited/bad flexibility
c) Unsuitable floor
d) Cold environment
e) Insufficient warm up
f) Insufficient cool down
g) New/difficult choreography
h) Different repertory
i) Repetitive movement
j) Partnering work
k) Incorrect technique/training

l) Ignoring early warning signs

m) Lack the sense of self-protection

n) Recurrence of old injury

o) Dance with an injury

p) Inadequate diet/hydration

q) Set/props

r) Costume/shoes

s) Rehearsal schedule

t) Other, please explain briefly__________________

20. What do you do if you suspect an injury? (multiple choice)

    a Seek professional medical treatment

    b Tell someone else

    c Take own preventative steps

    d Take pain killers

    e Continue to dance, but carefully

    f Ignore it

    g Hide it

    h Other, please specify_________

21. Who has the most influence in guiding your return to activity? (multiple choice)

    a Medical professional

    b Teacher

    c Yourself

    d Other, please specify___________

22. Did you have your dance class online in the period of COVID-19? Yes /no (single.)

23. If yes, how long have they been going on already? (single choice)

    a 1 months

    b 2 months

    c 3 months

    d 4 months

    e 5 months

    f 6 months

    g More than 6 months

24. What are you dancing on?

    a wooden floor

    b ceramic floor

    c dance floor (vinyl/foam/sprung)

    d Other, please specify_____________

25. Where did you do your exercise?

    a Bedroom

    b Living room

    c Your personal dance room in your home

    d Other, please specify_____

26. Did you get injured during this period time? Yes _ no_ (link with they had injury)? (single choice)

27. Which sites?

a Upper arm/forearm

b hands

c Elbows

d Wrists

e shoulders

f neck

g upper back

h Lower back

i Ribs

j Pelvis

k Hip joints

l Thighs

m Knees

n Lower legs

o Ankles

p Feet

28. If yes, what severity of your injury? (multiple choice)

    a Minor (can still dance fully)

    b Moderate (have to adapt their movement or can't do certain movements)

    c Severe (can't dance for at least 24 hours)

If severe, how long? ___days (single choice)

END OF SURVEY

Thank you for taking the time to complete this survey.
